# Pylephlebitis presenting as spontaneous coronary sinus thrombosis: a case report

**DOI:** 10.1186/s13256-017-1479-9

**Published:** 2017-11-02

**Authors:** Michael A. Hart, Mengistu A. Simegn

**Affiliations:** 1 General Internal Medicine, Hennepin County Medical Center, 701 Park Avenue, Minneapolis, MN 55415 USA; 2 Cardiology, Hennepin County Medical Center, 701 Park Avenue, Orange 5, Minneapolis, MN 55415 USA

**Keywords:** Coronary sinus, Thrombus, Transesophageal echocardiography

## Abstract

**Background:**

Coronary sinus thrombosis is a rare phenomenon. When identified, it most often is a complication of infective endocarditis or procedural intervention. We present an unusual and unreported case of spontaneous coronary sinus thrombosis as embolic sequela of an intra-abdominal infectious process.

**Case presentation:**

We report a case of a 61-year-old white woman with a history of end-stage renal disease on hemodialysis, paroxysmal atrial fibrillation not on long-term systemic anticoagulation, and history of recurrent diverticulitis that presented with acute onset abdominal pain and nausea. Computed tomography of her abdomen and pelvis with intravenous contrast was negative for acute intra-abdominal pathology, but incidentally identified an oval-shaped filling defect at the ostium of the coronary sinus suspicious for thrombus or mass which was confirmed on subsequent transesophageal echocardiogram. In light of her concomitant transaminitis but otherwise negative workup, the mass was believed to be thromboembolic in nature, originating within the hepatic venous system as a manifestation of recurrent diverticulitis with associated pylephlebitis and ultimately lodging into the coronary sinus. With the newly detected thrombus and history of paroxysmal atrial fibrillation, she was started on warfarin for therapeutic systemic anticoagulation that resolved her clot by 3-month follow up.

**Conclusions:**

Although coronary sinus thrombosis is rare, a high index of suspicion and close scrutiny of the venous system in patients with intra-abdominal infectious processes would prevent delay in management of this potentially serious complication. The discussion of this case highlights the anatomy of the cardiac venous system, the pathophysiology of thrombus formation, and the utility of transesophageal echocardiography in confirming a diagnosis and assessing treatment efficacy.

## Background

Coronary sinus thrombosis (CST) is an uncommon complication of instrumentation or procedures in the heart or great vessels. Here we present a case of spontaneous CST believed to be a manifestation of pylephlebitis with transient hepatic vasculature thrombus traveling to and lodging in the coronary sinus (CS). To the best of our knowledge, there are no reported cases of spontaneous CST as sequela of intra-abdominal infectious processes. The discussion highlights the anatomy of the cardiac venous system, the pathophysiology of thrombus formation, and treatment options for this rare but potentially serious and fatal condition.

## Case presentation

A 61-year-old white woman was hospitalized for acute onset abdominal pain and nausea. She had been well until 1 day earlier when she began to have continuous nausea, non-projectile recurrent emesis of ingested matter, anorexia, and epigastric abdominal pain.

Her past medical history included end-stage renal disease (ESRD) and two previous renal transplants secondary to immunoglobulin A (IgA) nephropathy on chronic immunosuppressive therapy with subsequent graft failure requiring return to hemodialysis for the past 3 years, non-ischemic dilated cardiomyopathy with nadir ejection fraction (EF) of 15 to 20%, hypertension, paroxysmal atrial fibrillation not on chronic systemic anticoagulation, mild to moderate non-obstructive coronary artery disease, and history of recurrent diverticulitis. She is unemployed, a former tobacco smoker quitting in 1993, she denied alcohol or recreational drug use, has no significant family history, and is allergic to angiotensin-converting enzyme (ACE) inhibitors (cough). Home medications include aspirin, atorvastatin, carvedilol, losartan, clonidine, and hydralazine.

On physical examination, she appeared acutely sick, afebrile, and normotensive with normal pulse oxygen saturation. A cardiovascular examination was significant for regular rate and rhythm with a 2/6 systolic murmur heard throughout the precordium. Pulmonary and neurologic examinations were unremarkable; extremity examination was notable for left upper extremity fistula with palpable thrill and mild pitting edema in bilateral lower extremities extending to mid-shin. Her abdomen was soft, mildly distended, and tender to palpation in the epigastrium without Murphy’s sign or rebound tenderness.

Hematology and chemistry panels were significant for neutrophilic leukocytosis (14.5 k/mm^3^), mild normocytic anemia (9.8 g/dL), hyponatremia (128 mEq/L), and hyperkalemia (6.1 mEq/L). Hepatic panel showed: transaminitis with aspartate aminotransferase (AST) 1078 and alanine aminotransferase (ALT) 660 IU/L; with normal total and fractional bilirubin; and mildly elevated alkaline phosphatase (113 IU/L). Evaluations for acetaminophen toxicity, infectious and autoimmune hepatitis, primary biliary cirrhosis, primary sclerosing cholangitis, and Wilson’s disease were unremarkable. Telemetry and electrocardiogram illustrated known paroxysmal atrial fibrillation.

**Fig. 1:**
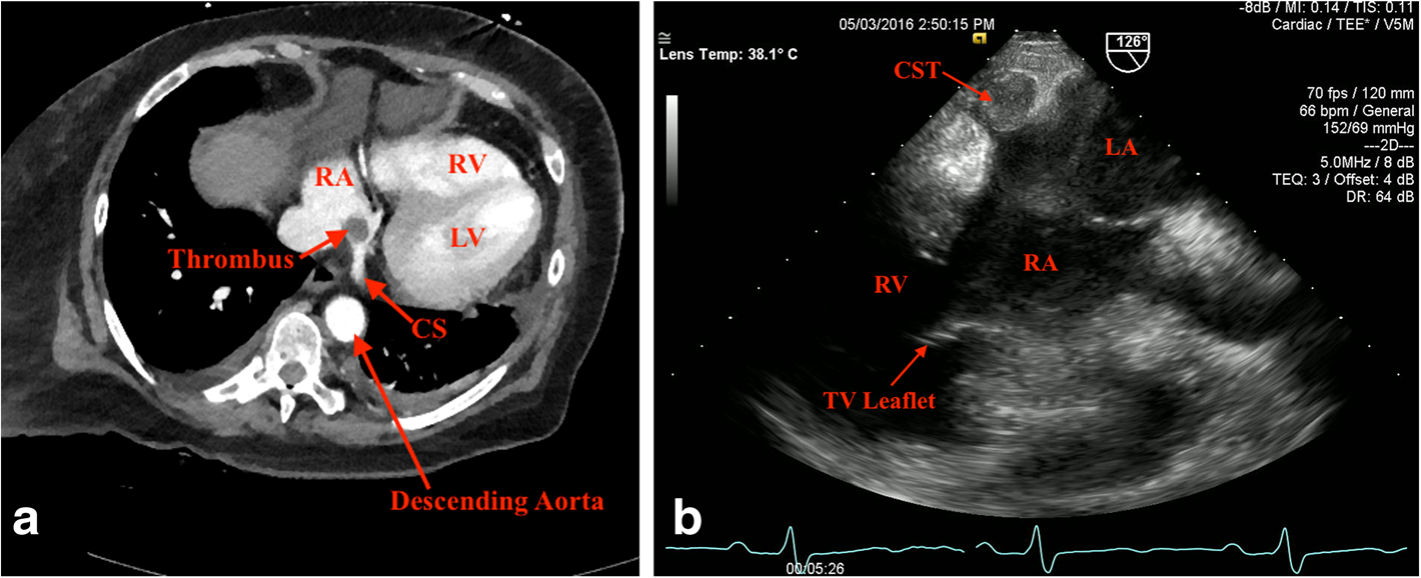
**a** Chest computed tomography with contrast at the level of coronary sinus showing an oval-shaped filling defect at the junction of the right atrium and coronary sinus suggestive of coronary sinus thrombus. **b** Transesophageal echocardiogram at the gastroesophageal junction positioned at 126° demonstrating a 1.5 × 1.5 cm echogenic globular mass at the ostium of the coronary sinus. *CS* coronary sinus, *CST* coronary sinus thrombus, *LA* left atrium, *LV* left ventricle, *RA* right atrium, *RV* right ventricle, *TV* tricuspid valve

Computed tomography (CT) of her abdomen and pelvis with intravenous contrast was negative for acute intra-abdominal pathology including thrombosis of the hepatic veins, but incidentally identified an oval-shaped filling defect at the junction of the right atrium and CS suspicious for thrombus or mass (Fig. 1a). Transesophageal echocardiogram (TEE), a better imaging modality for tissue characterization and assessment of the right atrium and CS, demonstrated a 1.5 × 1.5 cm echogenic globular mass at the ostium of the CS consistent with thrombus (Fig. 1b). The left ventricle was normal in size, but systolic function was mildly depressed.

**Fig. 2:**
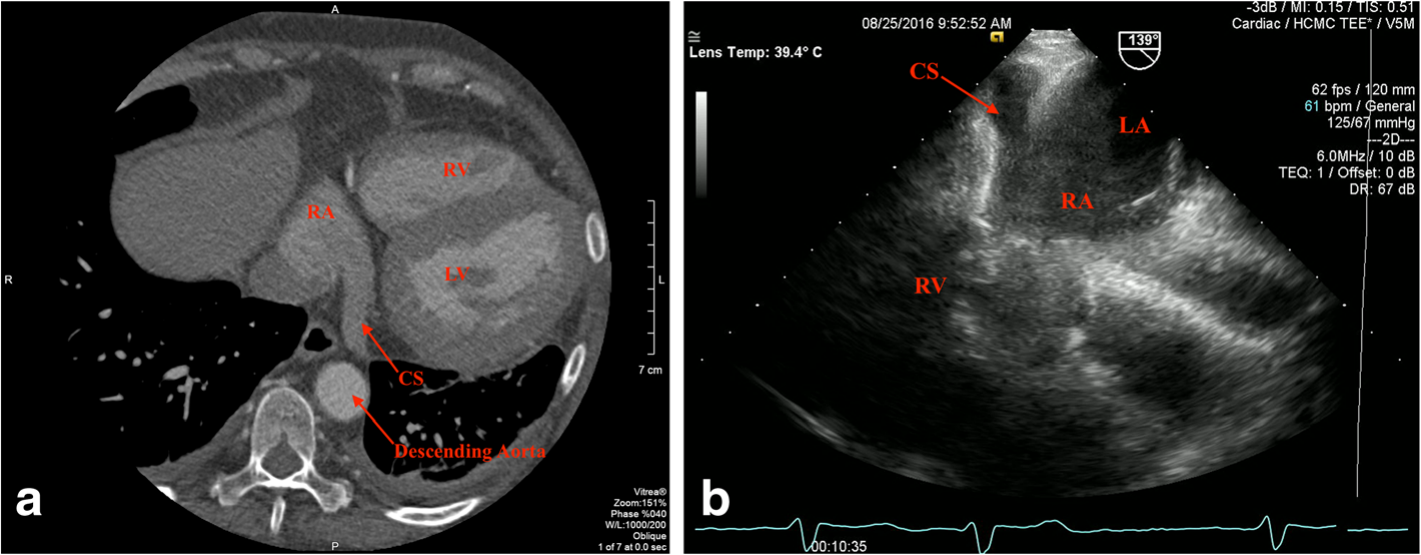
**a** Gated cardiac computed tomography at the coronary sinus level showing patent coronary sinus with resolution of the prior filling defect after 3 months of systemic anticoagulation. **b** Transesophageal echocardiogram at the gastroesophageal junction positioned at 139° confirmed patent coronary sinus. *CS* coronary sinus, *LA* left atrium, *LV* left ventricle, *RA* right atrium, *RV* right ventricle

With the newly detected thrombus and history of paroxysmal atrial fibrillation, our patient was started on warfarin for therapeutic systemic anticoagulation. At her 3-month out-patient visit, her liver enzymes had normalized, and TEE and gated cardiac CT, optimal imaging modality for assessment of the entire cardiac venous system, showed complete resolution of the CS thrombus (Fig. 2a and b). In light of paroxysmal atrial fibrillation and development of spontaneous CST, the decision was made at 6-month follow up to continue anticoagulation long term. Her subsequent clinical course was remarkable for development of polymicrobial intra-abdominal abscess (*Enterococcus faecalis, Escherichia coli*, and *Candida albicans*) 8 months after the index event requiring drain placement and 4 weeks of antibiotic therapy. She was ultimately discharged to sub-acute rehabilitation with plan for a partial colectomy to be done in the coming months after improvement in her functional capacity.

## Discussion

Here we present an unusual and unreported case of spontaneous CST as embolic sequela of an intra-abdominal infectious process. Thrombosis of the CS is an exceedingly rare event, with the majority of cases reported in the literature occurring in the setting of invasive procedures or infection. Development of CST in the absence of instrumentation is even less common, and believed to be secondary to underlying prothrombotic states [1–3]. Despite this, the potential complications of CST can be catastrophic and include myocardial infarction, cardiac tamponade, sudden cardiac arrest, and death [1, 4–6].

The CS is the largest venous collection system of the heart, accounting for over half of the total cardiac venous return. Its anatomical position at the posterior aspect of the coronary sulcus places it in close proximity to the inferior vena cava (IVC) through which it communicates directly with the rest of the body’s venous system. Our patient’s history of paroxysmal atrial fibrillation, ESRD, and chronic immunosuppressive therapy with recurrent diverticulitis is likely to have contributed to her overall inflammatory and procoagulant state. In this context, we suspect she developed congestive hepatitis from pylephlebitis and transient thrombosis of the hepatic vein, which then migrated to the right atrium and lodged in the CS ostium. This mechanism explains her presentation of acute abdominal pain, nausea, vomiting, transaminitis, and right atrial mass.

Pylephlebitis is a rare and serious complication of intra-abdominal infection and is most commonly associated with diverticulitis [7, 8]. Thrombotic communication between the hepatic and cardiac venous systems has been demonstrated in the literature on one other occasion, but again was in the setting of instrumentation [5]. Both mechanical and pharmacologic treatment of this condition have been successful in the past, with the latter strategy proving effective in resolving our patient’s CS thrombus and hepatitis [4, 9].

## Conclusions

Thrombus formation within the CS is extremely rare, with the majority of cases occurring as a result of instrumentation or prothrombotic states. Clinicians should be cognizant of the communication between the CS and the rest of the body’s venous system and the potential for serious complication when systemic thrombosis leads to embolization to the CS. Once identified, initiation of mechanical or pharmacologic treatment should follow promptly to avoid the potentially deadly manifestations of this phenomenon.

## Abbreviations

ACE: Angiotensin-converting enzyme
ALT: Alanine aminotransferase
AST: Aspartate aminotransferase
CS: Coronary sinus
CST: Coronary sinus thrombosis
CT: Computed tomography
EF: Ejection fraction
ESRD: End-stage renal disease
IgA: Immunoglobulin A
IVC: Inferior vena cava
TEE: Transesophageal echocardiogram
